# Creosote in Hospital Gangrene

**Published:** 1863

**Authors:** C. R. Parke

**Affiliations:** Bloomington, Ill.


ARTICLE X.

### CREOSOTE IN HOSPITAL GANGRENE.

BY C. R. PARKE, M.D., Bloomington, Ill.

TO THE McLEAN COUNTY MEDICAL SOCIETY:

MR. PRESIDENT:—I desire to lay before this Society a brief synopsis of my experience in hospital gangrene, which, I think, may be of service to many of my suffering countrymen now in the different hospitals of the land.

In the best regulated hospital buildings, erected with an eye particularly to ventilation and all that tends to the genial health of its inmates, hospital gangrene will frequently rage with unbridled fatality. During my services in the Crimean War, I had ample opportunity of testing the various modes of treating this troublesome and, too often, fatal disease. As every surgeon is aware of the symptoms characteristic of this disease, I shall not call your attention to them particularly, but to the principle point of interest,—the treatment.

During the late Crimean War, hospital gangrene and typhus fever were the principle diseases of the campaign. Many, in fact, most of the buildings used for hospital purposes during a military campaign are ill-adapted to such use, being incapable of thorough ventilation, and, of course, so much against the just merits of any treatment. Again, the Russian and German doctors, in the Russian service, were, for some reason or other, opposed to thorough ventilation, at least, when we (I mean the

American surgeons,) would return to the hospitals, we would invariably find the building closed securely at every point against the external atmosphere, with the exception of a small opening at the top of a few of the windows, about five inches in diameter, in which was placed a piece of tin freely perforated with holes. This was a very serious blunder, for which no surgeon could be excused, and one that contended vigorously against the success of any treatment.

During the winter and spring months particularly (1855–56), the weather was very unpropitious, being wet, damp, and foggy most of the time, with a profusion of mud in the streets. Of course, such a state of the atmosphere had a very depressing effect on the vital forces, and assisted greatly, in common with the vitiated atmosphere of an ill-ventilated hospital, in bringing about that peculiar state of the system favoring hospital gangrene. In the treatment of this disease, what seems to be called for? *First*, a pure atmosphere; *Second*, a disinfectant; *Third*, tonics and stimulants; and *Fourth, an external stimulating antiseptic.*

Of the first, it is necessary to say but little. As perfect a system of ventilation as is possible is imperiously demanded. Under the second head, we would say, a due regard to cleanliness is entirely necessary; at the sametime, let the bed-posts be wet frequently, and the floor sprinkled with a solution of chlo. lime. In the third place, use tonics and stimulants, wine, eggs, and soups, or stronger stimulants internally, if thought called for,—brandy, &c., &c. To fill the fourth indication, we have creosote, which acts, in most instances, like a charm. I apply it to the gangrene, stump, or wound directly, by means of a schwab, made by tying a small piece of sponge on the end of a stick. The application is severe, but not more so than the nitric acid, I doubt it being as severe. After being thoroughly rubbed over with the creosote, as above-described, I dress the parts with an ointment made of

℞. Creosote,..................................ʒi.

Oleum Olive,..................................Oj. M.

until the entire slough drops off, and the healthly red granulations are seen underneath. This will frequently take place in twenty-four hours after the first application. After this, it is only necessary to dress with some slightly stimulating ointment or wash. I have tried this treatment so thoroughly, MR. PRESIDENT, as not to leave the least possible doubt on my mind of its superiority over all other varieties of treatment in this disease.

